# Indeterminate Thyroid Nodules: From Cytology to Molecular Testing

**DOI:** 10.3390/diagnostics13183008

**Published:** 2023-09-20

**Authors:** Paola Vignali, Elisabetta Macerola, Anello Marcello Poma, Rebecca Sparavelli, Fulvio Basolo

**Affiliations:** Department of Surgical, Medical, Molecular Pathology and Critical Area, University of Pisa, Via Savi 10, 56126 Pisa, Italy; paola.vignali@phd.unipi.it (P.V.); marcello.poma@med.unipi.it (A.M.P.); rebecca.sparavelli@phd.unipi.it (R.S.)

**Keywords:** thyroid cytology, fine-needle aspiration (FNA), *RAS*-like, *BRAF*-like, molecular tests

## Abstract

Thyroid cancer is the most common malignancy of the endocrine system. Fine-needle aspiration (FNA) biopsy of thyroid nodules has become the gold standard procedure, in terms of cost and efficacy, for guiding clinicians towards appropriate patients’ management. One challenge for cytopathologists is to accurately classify cytological specimens as benign or malignant based on cytomorphological features. In fact, with a frequency ranging from 10% to 30%, nodules are diagnosed as indeterminate. In recent years, the mutational landscape of thyroid tumors has been extensively described, and two molecular profiles have been identified: *RAS*-like (*NRAS*, *HRAS,* and *KRAS* mutations; *EIF1AX* mutations; *BRAF K601E* mutation; and *PPARG* and *THADA* fusions) and *BRAF*^V600E^-like (including *BRAF*^V600E^ mutation and *RET* and *BRAF* fusions). The purpose of this review is to discuss the latest molecular findings in the context of indeterminate thyroid nodules, highlighting the role of molecular tests in patients’ management.

## 1. Introduction

Thyroid nodules are very common in the population, especially in women over 50 years of age [1,2]. Their identification is carried out through palpation, serum thyroid stimulating hormone (TSH) measurement, ultrasound (US) examination, and, if necessary, fine-needle aspiration (FNA) [3].

Ultrasound is a low-cost, non-invasive, and repeatable overtime exam that allows for an initial stratification of the risk of malignancy of the thyroid nodule. This classification, proposed by ACR, classifies thyroid nodules as benign, minimally suspicious, moderately suspicious, and highly suspicious for malignancy, based on the standard lexicon (TI-RADS) for ultrasound reporting. US features for the risk stratification are: hypoechogenicity, presence of microcalcifications, anteroposterior diameter larger than transverse diameter (taller-than-wide shape), irregular or lobulated margins. Therefore, thyroid nodules with a frankly benign US classification should not be subjected to FNA procedures [4,5].

With its low cost and high effectiveness, FNA biopsy became a standard procedure for thyroid nodule evaluation, guiding the management of patients toward surgical or conservative treatment. The cytological preparation is evaluated by experienced cytopathologists, who classify thyroid nodules based on one of the most widely established classification systems, e.g., the Italian Consensus for the Classification and Reporting of Thyroid Cytology (ICCRTC) and the Bethesda System for Reporting Thyroid Cytopathology (TBSRTC). These diagnostic systems include six categories, including non-diagnostic specimens and benign, indeterminate, suspicious, and malignant nodules. In most cases, benign and malignant classes show high diagnostic accuracy [6,7]. However, FNA has some limitations; in fact, approximately 70% of nodules are diagnosed as benign and 5–10% as malignant; therefore, the remaining 15–30% are classified as “indeterminate nodules” [8,9]. The differential diagnosis among indeterminate classes, composed mostly of follicular-architecture tumors (benign, pre-malignant, and frankly malignant), is very challenging [10]. Moreover, besides observer-related interpretation variability, in some cases, only one FNA could be fully representative of the nodule. In fact, a second FNA of the same nodule could help the pathologist make a more precise diagnosis. The experience of cytopathologists, added to the collaboration with clinicians, leads to better classification of thyroid nodules [11]. 

Over the last 10–15 years, the role of molecular analysis in improving the diagnosis and management of indeterminate nodules has been extensively investigated. The goal of this review is to collect and discuss the most recent molecular advances in the field of indeterminate thyroid cytology, highlighting how the application of molecular tests has changed the management of indeterminate nodules.

## 2. Cytological Classification Systems

Thyroid nodule aspiration is performed with a fine needle (from 23 to 27 gauge) under ultrasound guidance; the aspirated material is smeared on a glass slide or collected in specific solutions for liquid-based preparations; following alcoholic fixation (or simply after air-drying the slides), the May Grunwald Giemsa and/or Papanicolaou stains are recommended [3]. Moreover, the preparation can be observed by experienced cytopathologists, who will assign the appropriate cytological classification (Table 1) according to the observed cytomorphology.

The TIR1 category and Bethesda I both include non-diagnostic samples. In the Italian classification, this kind of sample could be inadequate and/or non-representative; in the first case, technical problems in the sample assembly occurred; in the second case, the cells are absent or insufficient for a definitive diagnosis. According to the third edition of TBSRTC, this classification includes FNA that should show cells well-preserved, well-stained, and easily visualized, but also inadequate samples with obscuring blood, poor cell preservation, and an insufficient presence of follicular cells, which by definition contain non-diagnostic information [7]. The Italian classification also includes the possibility of TIR1C (non-diagnostic cystic), which is the diagnostic class for samples obtained from cystic lesions, showing a cellularity that does not meet the requirements for adequacy. The risk of malignancy (ROM) of TIR1 is difficult to define, while the ROM of TIR1C should be low (variable based on clinical findings). The ROM for Bethesda I, calculated among surgically excised nodules initially classified as non-diagnostic, ranges from 5 to 20% (average, 13%). [6,7]

There are no differences between TIR2 and Bethesda II; both include non-malignant/benign lesions. Thyroid nodules belonging to these categories include colloid goiter, hyperplastic nodules, autoimmune and granulomatous thyroiditis, and other non-neoplastic conditions. Suggested actions are clinical and sonographic follow-up; furthermore, repeat FNA is necessary in cases of nodule growth or structural changes. The ROM of TIR2 should be less than 3%, while the ROM of Bethesda II nodules is in a range from 2 to 7% (average, 4%). 

**Figure 1:**
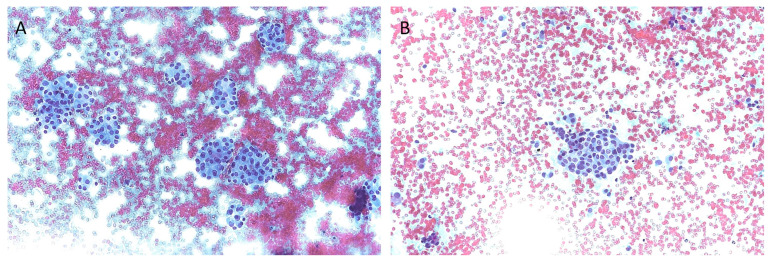
Cytologic images of indeterminate thyroid nodules. (**A**): microscopic image of a low-risk indeterminate lesion (TIR3A): groups of follicular cells organized in micro-follicular structures. The microfollicles are composed of cells without nuclear and/or cytoplasmic atypia (200 original magnification). (**B**): microscopic image of a high-risk indeterminate lesion (TIR3B): group of follicular cells with mild nuclear atypia. Some nuclei show hyperchromasia, and some nuclei have an elongated appearance. No elements strongly indicative of papillary carcinoma (i.e., nuclear presudo-inclusions and/or nuclear grooves) are present (200 original magnification).

The TIR3A category includes Low-Risk Indeterminate Lesions (LRIL, Figure 1A), and the corresponding Bethesda III category includes nodules with Atypia of Undetermined Significance (AUS). TIR3A lesions are characterized by a microfollicular pattern in a background of poor colloid amount, but the appearance of microfollicles is not sufficient to suspect a follicular neoplasm. The AUS category contains cases with “nuclear” and “other” atypia. The term “other” refers to cases with architectural atypia, oncocytic atypia, and lymphocytic atypia. For Bethesda III, the new TBSRTC discontinues the term “follicular lesion of undetermined significance” (FLUS). In TIR3A nodules, repeat FNA and clinical follow-up are recommended, while in Bethesda III nodules, molecular testing or lobectomy are also recommended. The ROM of these categories should be less than 10% for TIR3A, compared with 13–30% (average, 22%) of AUS [6,7]. The TIR3A and Bethesda III categories do not show perfect overlapping; the reason is a different interpretation of nuclear atypia. The latter are not included in the TIR3A sub-category, while in the Bethesda system, nodules showing focal and/or mild nuclear atypia can be included in the low-risk indeterminate category (Bethesda III) [12].

The TIR3B category includes high-risk indeterminate lesions (HRIL, Figure 1B), while the Bethesda IV category includes follicular neoplasm (FN). TIR3B specimens show high cellularity, repetitive microfollicular/trabecular arrangement, and poor or absent colloid, suggesting a follicular neoplasm. Moreover, this category also includes mild nuclear alterations typical of papillary carcinoma (PTC), which are too focal or mild for a call of suspicious nodules. The ROM can reach about 30%. The FN category includes follicular-pattered nodules with mild nuclear changes, but true papillae and intranuclear pseudo-inclusion are absent. The ROM ranges from 23 to 34% (average, 30%). The new TBSRTC recommends not using the term “suspicious for follicular neoplasm” (SFN) to avoid generating confusion with the Bethesda V category. Suggested actions for these categories are molecular testing and surgery (mostly diagnostic lobectomy) [6,7].

The risk of malignancy observed in indeterminate categories sometimes deviates, even significantly, from the expected ROM indicated by the classification systems. This discordance could be explained by a “selection bias”: the ROM is calculated considering the histological outcome of nodules; therefore, only patients who underwent surgery are included since the histological diagnosis is known [13]. According to a recent meta-analysis conducted on indeterminate thyroid nodules diagnosed according to the Italian system, the ROM of TIR3A nodules was 12.4%, while in TIR3B, the cancer prevalence was as high as 44.4% [14]. In addition, in clinical practice, more than half of indeterminate nodules are surgically treated, contrary to the indications of classification systems [13,14]. In fact, surgical rates among TIR3A and TIR3B nodules reach up to 48.3% and 75.2%, respectively [14].

The TIR4 and TIR5 categories, as well as Bethesda V and VI, include lesions suspicious for malignancy (SUS) and malignant lesions. Specimens with suspicion of malignancy show low cellularity and normal and atypical cells without obvious malignant features. Surgery is recommended, and the risk of malignancy is very high (60–80% and more than 95% for TIR4 and TIR5, respectively, and an average of 74% with a range of 67–83% in Bethesda V). In nodules diagnosed as malignant lesions, a precise indication of the tumor type (papillary carcinoma and its subtypes, medullary, high-grade follicular-derived thyroid carcinoma, anaplastic carcinoma) is not always possible based on cyto-morphologic appearance. The correlation with clinical data is crucial in these cases. Surgery is recommended since the risk of malignancy could be around 99% for TIR5 and an average of 97% (ranging from 97% to 100%) for Bethesda VI [6,7].

Regarding non-invasive follicular neoplasm with papillary-like nuclear features (NIFTP), surgical diagnosis is necessary. Most NIFTPs are cytologically classified as indeterminate nodules, and only a small part is included among the suspicious and malignant categories. They are not considered benign nor malignant tumors, but a separate histological category [15]. In the new WHO classification of tumors of endocrine organs, NIFTPs are placed in the category of “low-risk neoplasms” [16]. In literature, it is not unusual to calculate the ROM of FNA classes in two ways: including and not including NIFTP among malignant lesions.

## 3. Biomarkers in Indeterminate Thyroid Nodules

A range from 15 to 20% of thyroid nodules are classified as indeterminate; the histological diagnosis of these nodules includes benign tumors, low-risk, and malignant neoplasms, including follicular and papillary thyroid cancer [3].

**Figure 2:**
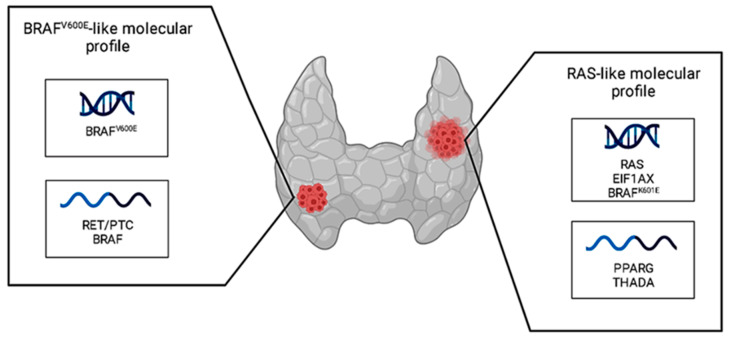
Schematic representation of *BRAF*^V600E^-like and *RAS*-like molecular profiles. On the left, the *BRAF*^V600E^-like molecular profile is characterized by the *BRAF*^V600E^ mutation and RET/PTC and BRAF fusions. On the right, the *RAS*-like molecular profile is characterized by *NRAS*, *HRAS*, *KRAS*, *EIF1AX,* and *BRAF^K601E^* mutations and *PPARG* and *THADA* fusions. Created with BioRender.com accessed on 4 July 2023.

In the last 10 years, there has been much interest among the scientific community regarding the molecular landscape of thyroid cancer. In fact, in 2014, the Cancer Genome Atlas Research Network (TGCA) studied the molecular profile of 496 papillary thyroid cancers (PTCs), mainly classical and follicular variants, and delivered two distinct molecular profiles: *BRAF^V600E^*-like and *RAS*-like [17]. As shown in Figure 2, the first included the *BRAF^V600E^* mutation and *RET/PTC* and *BRAF* fusions, while the second included *RAS* family (*NRAS*, *HRAS,* and *KRAS*) mutations, *EIF1AX* mutations, the *BRAF^K601E^* mutation, and *PPARG* and *THADA* fusions. 

The *BRAF* gene encodes a cytoplasmic serine/threonine kinase (BRAF), while RAS proteins (NRAS, HRAS, and KRAS) are small GTPases, and both are core components of the RAS-RAF-MEK-ERK pathway (MAPK pathway). Under physiological conditions, the MAPK pathway results in the activation of several cytoplasmic and nuclear proteins that promote cell proliferation, differentiation, senescence, and apoptosis; therefore, it regulates important cell functions [18,19]. On the other hand, several oncogenic mutations occurring in *BRAF*, *NRAS*, *HRAS,* and *KRAS* implicate constitutive MAPK pathway activity, resulting in hyperproliferative developmental disorder and tumorigenesis [20,21].

*RET* rearrangements first described in papillary thyroid carcinoma are generally known as “*RET/PTC*”, of which the most frequently detected are *RET/PTC1* and *RET/PTC3*. RET/PTC1 is a chimeric protein generated by the fusion of the 3′ *RET* tyrosine-kinase domain and 5′ terminal region of the *CCDC6* gene; RET/PTC3 is a fusion transcript generated by the *RET* and *NCOA4* genes. *RET/PTC* fusions are characterized by a high capability to generate a coiled-coil domain, resulting in the constitutive dimerization and activation of the RET thyrosin-kinase domain, promoting cell growth, differentiation, proliferation, and survival [22,23].

The *EIF1AX* gene encodes for eukaryotic translation initiation factor 1A. The X-chromosomal (eIF1A) protein and its mutations interfere in global protein translation. *EIF1AX* mutations, in the same way as *RAS* mutations, can be detected both in benign and malignant thyroid neoplasms. However, the coexistence of *EIF1AX* and *RAS* mutations has been described in poorly differentiated and anaplastic carcinomas, so it seems related to tumor aggressiveness [24].

The *PPARG* gene encodes the peroxisome proliferator-activated receptor gamma (PPARG) protein, which is a nuclear receptor functioning as a transcription factor. In thyroid cancer, the *PPARG* gene is rearranged with *PAX8*, a gene encoding for a transcription factor required for thyroid differentiation. PAX8/PPARG is a chimeric oncogenic protein that promotes cell growth and reduces the rate of apoptosis [25,26].

Finally, the *THADA* gene encodes thyroid adenoma-associated protein (THADA). In thyroid cancer, it is rearranged with insulin-like growth factor 2 mRNA-binding protein 3 (IGF2BP3), and this fusion determines high expression of the IGF2BP3 protein, resulting in a deregulated activation of MAPK and PI3K signaling [27,28].

From a morphological point of view, the *BRAF*-like profile is characterized by papillary architecture with lower expression of the genes related to thyroid differentiation, while the *RAS*-like profile is characterized by follicular architecture with normal expression of the genes related to thyroid differentiation [29]. 

Moreover, it is known that, in addition to the above-described mutually exclusive driver mutations, secondary mutations in the *TERT* promoter and *TP53* genes may identify thyroid cancers with a higher risk of tumor recurrence and disease-specific mortality [17,30,31,32]. 

Telomerase reverse transcriptase protein (TERT) is the catalytic subunit of telomerase. The role of the telomerase enzyme is to lengthen telomers to allow cell replication without loss of genetic material. In thyroid cancer, the presence of *TERT* promoter mutations determines the creation of new binding sites for a family of transcription factors, ultimately leading to an upregulation of telomerase transcription. Thanks to this mechanism, cells can avoid senescence and death [33]. 

The *TP53* gene encodes for the p53 protein, which plays an important role in cell cycle regulation. In detail, p53 can trigger cell cycle arrest, DNA repair, senescence, apoptosis, and autophagy. For this reason, in cancer, mutations in the *TP53* gene usually cause protein loss or non-functioning/truncated protein forms, promoting cell survival and proliferation [34]. Table 2 summarizes the key role of altered biomarkers detectable in indeterminate thyroid nodules.

## 4. Molecular Testing and Indeterminate Thyroid Nodules

From the TGCA study, the *BRAF*-like and *RAS*-like molecular profiles seem to be associated with PTCs, classical subtypes, and follicular subtypes, but subsequently, these two distinct molecular profiles could be extended to other thyroid tumor histotypes. For instance, NIFTP and follicular thyroid carcinoma can be considered *RAS*-like tumors; moreover, benign tumors can also show a *RAS*-like molecular profile [35]. The rate of malignancy of *RAS*-like tumors can vary widely based on geography, institutional, and pathological differences in the threshold for cancer diagnosis [36].

In 2019, the Nikiforov group analyzed 257 indeterminate nodules with ThyroSeq v3 GC, which is a targeted next-generation sequencing test examining selected regions of 112 thyroid cancer-related genes for point mutations, insertions/deletions (INDELs), gene fusions, copy number alterations, and gene expression alterations. They demonstrated that nodules with mutations are malignant in histology, but they are a very small part (<10%) of indeterminate nodules. *BRAF^V600E^* is the most common mutation in thyroid cancer, with a high frequency (35–65%) in PTC [37]. Indeterminate nodules have a malignancy risk ranging from 20% to 30%, and less than 5% of these are positive for *BRAF^V600E^* [38,39]. The remaining part of indeterminate nodules, negative for the *BRAF^V600E^* mutation, remains an area of interest and ongoing study; for the most part, they are benign nodules, follicular adenomas, or tumors with a follicular pattern, thus having a *RAS*-like mutational profile. The presence of *RAS*-like mutations confirms the presence of a clonal process but cannot discriminate between benign, malignant, or low-risk lesions, such as NIFTP [40].

In practice, the detection of an indeterminate *RAS*-like nodule does not necessarily prompt surgical intervention. Based on the clinical characteristics of the patient, a diagnostic lobectomy can be performed, followed by a histological diagnosis, which will determine the definitive management of the patient. According to the histological tumor type, but also the degree of local invasion and possibly lymph node metastasis, a complete thyroidectomy can be considered, and based on the clinical recommendations, radioiodine therapy may be administered. If the nodule is histologically benign or NIFTP, the patient can be considered cured [41].

To address the issue of overtreating patients with indeterminate nodules, the scientific community has mobilized to find molecular tests aimed at better stratifying the risk of malignancy within this cytological category. In the context of indeterminate nodules, there are “rule-in” and “rule-out” molecular tests. The term “rule-in” refers to those tests capable of identifying malignant nodules and suggesting surgical treatment. On the other hand, the term “rule-out” refers to those tests capable of identifying benign nodules and thus suggesting clinical follow-up rather than surgery. A rule-out test should have high sensitivity and negative predictive value (NPV). On the contrary, a good rule-in test should have high specificity and positive predictive value (PPV) [42]. 

The currently available molecular tests for indeterminate nodules range from small panels, such as the 7-gene panel, including *BRAF*, *NRAS*, *KRAS*, *HRAS*, *RET/PTC1*, *RET/PTC3,* and *PPARG/PAX8*, to panels containing a higher number of genes. The 7-gene panel includes the most common alterations detectable in thyroid nodules; thus, it could be a useful “rule-in” test, as it shows high specificity. This test can considerably increase the ROM of indeterminate nodules; in fact, the probability of cancer before and after the test goes from 16% to 67% [43]. In an Italian multicentric study, it was reported that the specificity and PPV of the 7-gene panel were higher in Bethesda IV compared to Bethesda III nodules (90% versus 64% specificity and 80% versus 42.6% PPV) [44]. Independently from the used molecular panel, while *BRAF^V600E^* mutations and *RET/PTC* rearrangements are highly predictive of malignancy (with over 95% ROM), the risk of malignancy for mutated *RAS* genes ranges between 57% and 87% [45]. In this context, it has been demonstrated that by combining molecular testing results with ultrasound features of nodules (EU-TIRADS system), the risk stratification of indeterminate nodules can be significantly improved [46]. 

Commercial panels for molecular testing of indeterminate thyroid nodules include Thyroseq v3, Afirma GSC, and ThyGeNEXT. Thyroseq v3 is a wide panel, including 112 genes. It has shown good results in terms of NPV, therefore, as a “rule-out” test, but compared to its previous version, it does not confirm its performance as a “rule-in” test. Afirma GSC is currently the only available diagnostic test that combines next-generation RNA sequencing with the detection of small genomic alterations not detectable by conventional methods. Afirma GSC has improved its “rule-out” testing capabilities compared to the previous version, but its “rule-in” test proprieties have not yet been met [42]. ThyGeNEXT is a NGS panel aimed at sequencing five genes (*BRAF*, *NRAS*, *HRAS*, *KRAS,* and *PIK3CA*) and three types of fusions (*RET/PTC1*, *RET/PTC3,* and *PPARG/PAX8*), combined with ThyraMIR, which measures the expression level of 10 microRNAs (miRNAs), increasing the efficiency of ThyGeNEXT.

Molecular tests focused on TERT promoter mutations and TP53 gene mutations are not widely used in the indeterminate nodule category. The reason is that the presence of mutations in these two genes is associated with aggressive histological phenotypes, which are usually not classified in the categories with indeterminate cytology. Therefore, the frequency of TERT promoter and TP53 mutations in indeterminate nodules is very low [47,48].

Over the last few years, miRNA expression profiles have been investigated with the aim of improving the pre-surgical distinction between benign and malignant lesions, even in the presence of *RAS*-like alterations. MiRNAs are small, non-coding RNAs approximately 19–25 nucleotides in length. The role of miRNA is to regulate gene expression by inactivating or destroying messenger RNA (mRNA). miRNA can act both as oncogenic miRNA (oncomiR) and as tumor suppressor miRNA; in cancer, oncomiR is often upregulated, while the expression of tumor suppressor miRNA can be downregulated [49].

Cipriani et al. performed a retrospective evaluation of 140 FNAs, of which 92.2% had indeterminate cytology, testing the next-generation sequencing panel ThyGeNEXT combined with the miRNA expression algorithmic profile ThyraMIR in case of a wild-type result or positivity for an alteration with weak positive predictive value, i.e., *RAS*-like alterations. They concluded that thyroid nodules with *RAS*-like mutations and a negative miRNA profile have a low risk of malignancy and generally represent indolent follicular neoplasms, unless accompanied by other molecular findings such as *TERT* promoter mutations [29]. 

Regarding indeterminate thyroid nodules negative for *BRAF* or *RAS* mutations, Macerola and colleagues performed a retrospective search of indeterminate FNAs with available histology. Specifically, they analyzed the miRNA expression profile by using the nCounter system directly on cytological slides of nodules corresponding to follicular adenomas (FAs), follicular-variant PTCs, and NIFTPs, with the aim of finding miRNAs able to differentiate benign from malignant follicular-architecture lesions. They discovered a significant downregulation of *miR-7-5p* and *miR-548ar-5p* in *BRAF-* and *RAS*-negative FVPTCs compared to FAs [50]. The same research group expanded the analysis of miRNA to investigate differences between follicular-patterned tumors positive and negative for *RAS* mutations. They identified 12 deregulated miRNAs between *RAS*-mutated and *RAS*-negative FAs. Additionally, in the comparison between benign and malignant tumors positive for RAS mutations, three particular miRNAs of interest emerged: *miR-146b-5p*, which showed increasing upregulation from benign nodules to *RAS*-positive malignant nodules (i.e., *miR-144-3p* and *miR-451a)*, which were downregulated in malignant *RAS*-mutated tumors [51].

## 5. Conclusions

Indeterminate thyroid nodules remain a challenge in cytological practice. Cytological morphology alone cannot resolve the uncertainty around indeterminate thyroid nodules; clinical evaluation is paramount in the management of these patients, but often only a diagnostic surgery followed by a tissue evaluation can define the nature of the lesion. A molecular investigation could help in the pre-surgical management of thyroid nodule patients. The use of simple molecular tools, such as the seven-gene panel, is becoming more and more widespread; moreover, many efforts have been made toward the refinement of the risk of malignancy in nodules carrying *RAS*-like alterations. The continuous improvement of molecular tests and the efforts to make them more and more accurate and less expensive will likely make them part and parcel of routine indeterminate thyroid nodule evaluation.

## Figures and Tables

**Table 1:** Comparison between ICCRTC and TBSRTC.

The Italian Consensus for the Classification and Reporting of Thyroid Cytology (ICCRTC)			The Bethesda System for Reporting Thyroid Cytopathology (TBSRTC)		
Class	Diagnostic category	Risk of malignancy (%)	Class	Diagnostic category	Risk of malignancy (%)
TIR1	Inadequate and/or non-representative	Not defined	I	Non-diagnostic samples	13
TIR1C	Non-diagnostic cystic	Low			
TIR2	Non-malignant/benign lesions	<3	II	Benign lesions	4
TIR3A	Low-risk indeterminate lesions	<10	III	Atypia of undetermined significance (AUS)	22
TIR3B	High-risk indeterminate lesions	30	IV	Follicular neoplasm (FN)	30
TIR4	Suspicious for malignancy	60–80	V	Suspicious for malignancy	74
TIR5	Malignant lesions	>95	VI	Malignant lesions	97

**Table 2:** Key role of the most frequent biomarkers altered in indeterminate thyroid nodules.

Gene	Protein	Role of Molecular Markers Alterations
*BRAF*	BRAF	Mutations in these proteins implicate constitutive MAPK pathway activity, resulting in hyperproliferative developmental disorder, leading to tumorigenesis.
*RAS* family: *NRAS* *HRAS* *KRAS*	RAS family: NRAS HRAS KRAS	
*CCDC6::RET*	RET/PTC1	This rearrangement results in a constitutive dimerization and activation of the RET thyrosin-kinase domain, promoting cell growth, differentiation, proliferation, and survival.
*NCOA4::RET*	RET/PTC3	
*EIF1AX*	eIF1A	Mutations in this eukaryotic translation initiation factor 1A interfere with global protein translation.
*PAX8::PPARG*	PAX8/PPARG	This rearrangement generates a chimeric oncogenic protein, which promotes cell growth and reduces the rate of apoptosis.
*THADA::IGF2BP3*	THADA/IGF2BP3	This rearrangement implicates a high expression of the IGF2BP3 protein, resulting in a deregulated activation of MAPK and PI3K signaling.
*TERT* promoter	TERT	*TERT* promoter mutations implicate an upregulation of telomerase transcription, avoiding senescence and apoptosis.
*TP53*	p53	Mutations in *TP53* genes cause protein loss or non-functioning/truncated protein forms, promoting cell survival and proliferation.

## Data Availability

No new data were created during the writing of this review.
